# Periapical Microsurgery with an Endoscope and Microscope of Two Upper Central Incisors Already Subjected to Periapical Surgery 25 Years Ago

**DOI:** 10.1155/2020/8885568

**Published:** 2020-12-01

**Authors:** Pablo Glera-Suarez, Blanca Serra-Pastor, David Peñarrocha-Oltra, Miguel Peñarrocha-Diago, Cosme Gay-Escoda

**Affiliations:** ^1^Department of Stomatology, Faculty of Medicine and Dentistry, University of Valencia, Spain; ^2^Prosthodontics Unit, Department of Stomatology, Faculty of Medicine and Dentistry, University of Valencia, Spain; ^3^IDIBELL Institute, Barcelona, Spain; ^4^Oral and Maxillofacial Surgery Department, Faculty of Medicine and Health Sciences (School of Dentistry), University of Barcelona, Spain

## Abstract

**Introduction:**

The present clinical case describes periapical microsurgery with an endoscope and microscope in a patient already treated 25 years ago due to persistent periapical disease of the two central upper incisors, restored with poorly adapted crowns. *Clinical Case*. The first periapical surgery had been performed with silver amalgam as a retrograde filler material, causing grayish staining of the buccal mucosa. Periapical surgery was performed raising a submarginal flap with ostectomy and apicoectomy, retrograde cavity preparation with ultrasound tips, and filling with mineral trioxide aggregate (MTA). After soft tissue healing and complete bone regeneration of the lesion, retreatment of the incisors with a fixed prosthesis was carried out, adopting the biologically oriented preparation technique (BOPT).

**Conclusions:**

The described periapical microsurgery approach with magnification and illumination of the surgical field was found to be effective, avoiding the need to extract the two central upper incisors.

## 1. Introduction

Important advances have been made in periapical surgery, including the introduction of surgical field illumination and magnification with a microscope or endoscope and the use of high-quality filler materials. The aim of retrograde filling is to secure good sealing of the cavity and the accessory canals and dentinal tubules—avoiding bacterial microleakage in order to ensure success of the surgical technique [1]. In the period between 1940 and 2000, silver amalgam was the most widely used retrograde filler material. However, since the year 2000, different alternative materials have been introduced, such as glass ionomers, zinc oxide-eugenol cements, and gold or mineral trioxide aggregate (MTA) [2]. The properties of these newer materials have been studied in order to determine which is the best option for use in periapical surgery. In this regard, MTA has been shown to be the most stable material over the long term, minimizing leakage, with lesser inflammation of the periapical tissues and with a higher treatment success rate than the rest of the materials [3–8]. The development of ultrasound tips for retrograde cavity preparation, together with the incorporation of microsurgery (field amplification with an endoscope and illumination and magnification with a microscope), has allowed periapical surgery to become a predictable solution for the management of persistent chronic periapical periodontitis, with a success rate of approximately 92% [2].

In the event of persistent chronic periapical periodontitis affecting an incisor already previously subjected to periapical surgery that has failed, a possible solution is to remove the tooth and place a dental implant—though this involves more aggressive surgery, with greater morbidity and higher costs for the patient.

Endodontic microsurgery with the use of new filler materials, ultrasound tips, and magnification techniques [9] has increased the treatment success rates. We thus decided to perform periapical microsurgery with an endoscope and microscope in a patient already treated 25 years ago with silver amalgam cavity filling due to persistent periapical disease of the two central upper incisors. Our approach avoided removal of the incisors, and retreatment of the incisors was completed with a fixed prosthesis, adopting the biologically oriented preparation technique (BOPT).

## 2. Case Presentation

**Figure 1:**
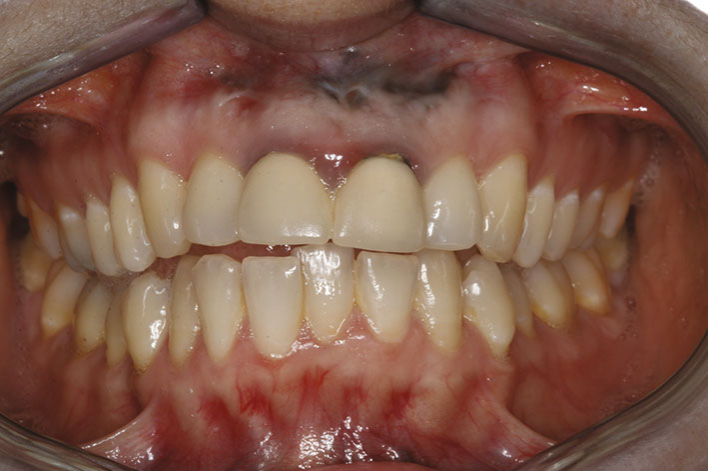
Preoperative clinical view. Note the reddening and inflammation at the buccal level of 1.1 and 2.1 and of the interdental papilla, as well as grayish buccal and apical staining of the central incisors, caused by the presence of the silver amalgam filler material.

**Figure 2:**
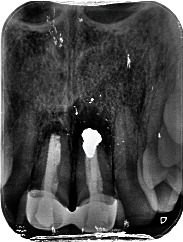
Preoperative periapical radiograph showing a radiolucent image around the root of 1.1 and a larger radiolucency around 2.1.

**Figure 3:**
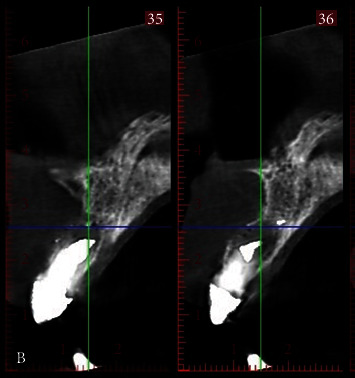
Preoperative sagittal CBCT view showing destruction of the buccal cortical layer of 2.1.

**Figure 4:**
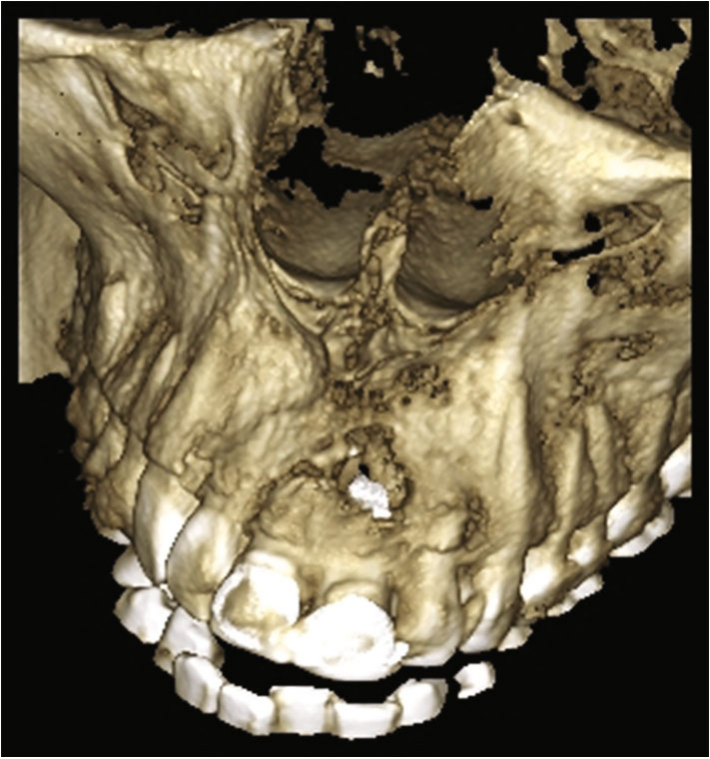
Preoperative three-dimensional radiographic reconstruction showing destruction of the buccal cortical layer of 2.1.

A 54-year-old woman who had undergone periapical surgery 25 years ago to solve persistent chronic periapical periodontitis of the two central upper incisors reported with discomfort in the periapical zones of the mentioned teeth. The intraoral exploration revealed reddening and inflammation at the buccal level of 1.1 and 2.1 and of the interdental papilla, as well as grayish buccal and apical staining of the central incisors, caused by the presence of the silver amalgam filler material (Figure 1). The periapical radiographs revealed radiolucencies around the apexes of 1.1 and 2.1 (Figure 2). Cone beam computed tomography (CBCT) (Planmeca®, Helsinki, Finland) evidenced bone destruction at 1.1 (Figure 3) and 2.1, with the involvement also of the buccal cortical layer of 2.1 (Figure 4).

**Figure 5:**
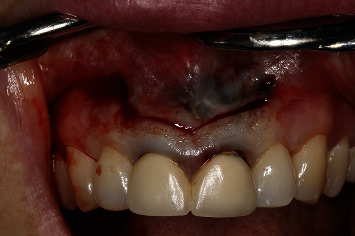
Submarginal incision.

**Figure 6:**
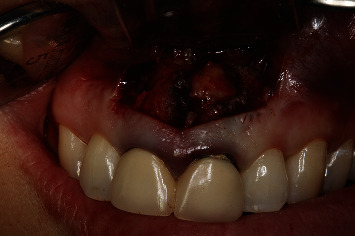
Raising of the full-thickness mucoperiosteal flap.

Periapical surgery of the affected teeth was carried out. Infiltration anesthesia was performed in the vestibular depth of both teeth using two carpules of 4% articaine with epinephrine 1 : 100,000 (Inibsa®, Lliça de Vall, Spain). Magnification was provided by an EXTARO 300 dental microscope (Zeiss®, Oberkochen, Germany). A number 15C scalpel was used to perform a horizontal submarginal incision (Figure 5) at 3-4 mm from the gingival margin, with vertical releasing incisions distal to 1.2 and 2.2 and the raising of a full-thickness mucoperiosteal flap (Figure 6).

**Figure 7:**
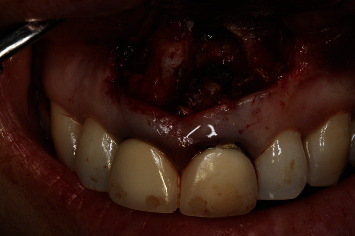
Ostectomy and curettage of the periapical lesions of 1.1 and 2.1.

**Figure 8:**
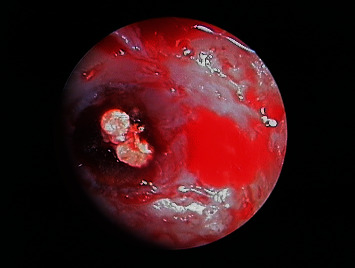
Endoscopic view of 1.1 before removal of the old filler material.

**Figure 9:**
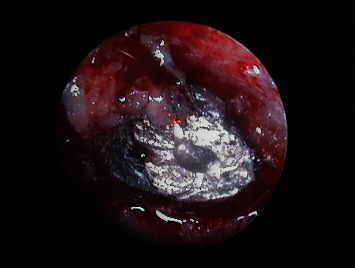
Endoscopic view of 2.1 before removal of the old filler material.

**Figure 10:**
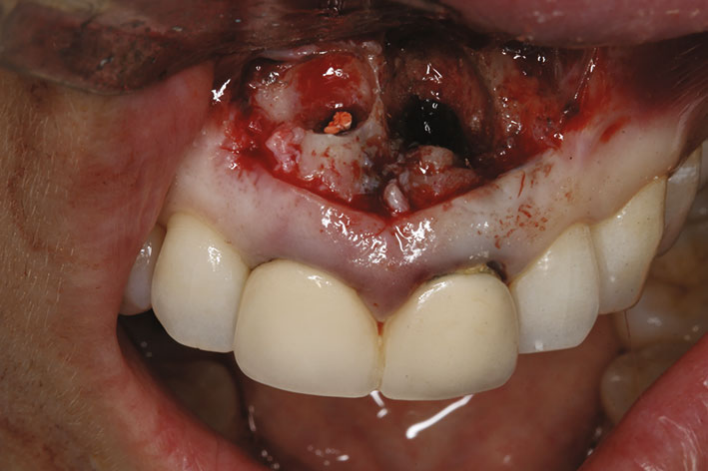
Clinical view of the removal of the old filler material of 1.1 and 2.1.

An ostectomy was performed for full visualization of the lesion, using a 6-blade tungsten carbide drill mounted in a handpiece with abundant sterile saline irrigation. The periapical inflammatory tissue was removed for posterior histopathological study (Figure 7), and inspection of the roots of 1.1 (Figure 8) and 2.1 (Figure 9) was made using a rigid endoscope (Karl Storz®, Tuttlingen, Germany), with the application of methylene blue dye to the zone in order to discard possible root fractures. The silver amalgam was removed from the retrograde cavities using ultrasound tips (Piezomed®, W&H, Bürmoos, Austria) (Figure 10).

**Figure 11:**
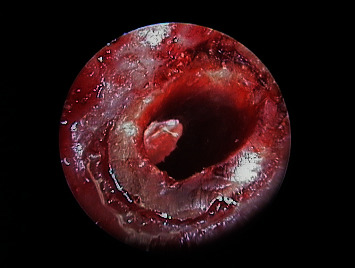
Endoscopic view of 1.1 after removal of the old filler material and checking of retrograde cavity integrity.

**Figure 12:**
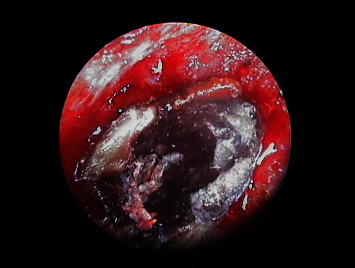
Endoscopic view of 2.1 after removal of the old filler material and checking of retrograde cavity integrity.

**Figure 13:**
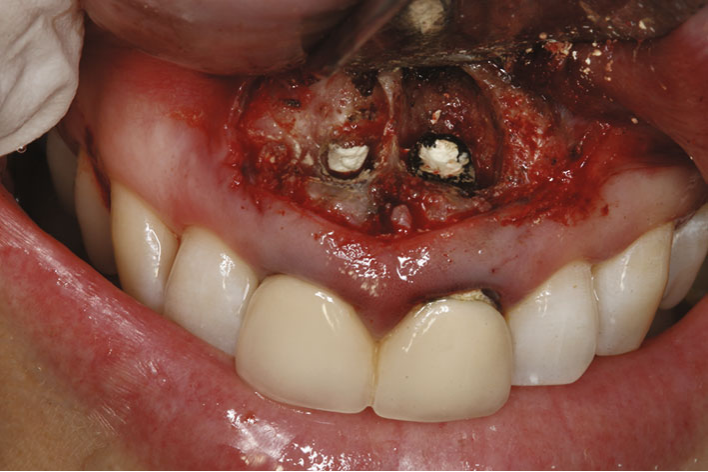
Clinical view of filling with MTA of the retrograde cavities of 1.1 and 2.1.

**Figure 14:**
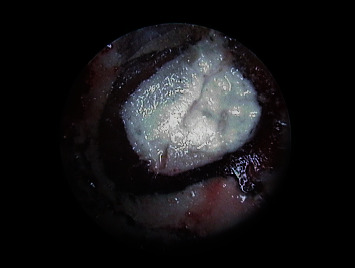
Endoscopic view of 1.1 after filling of the retrograde cavity with MTA.

**Figure 15:**
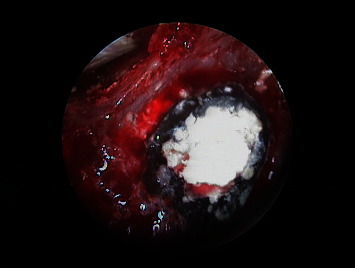
Endoscopic view of 2.1 after filling of the retrograde cavity with MTA.

**Figure 16:**
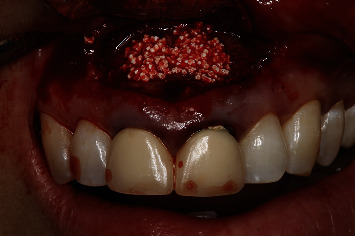
Regeneration of the defect with a synthetic bone graft.

**Figure 17:**
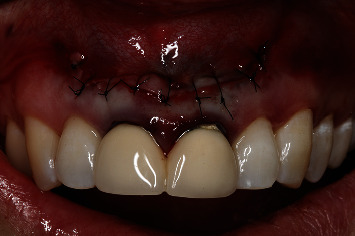
Flap suture with a non-reabsorbable 4/0 multifilament suture.

The endoscope was used to check that the retrograde cavities of 1.1 (Figure 11) and 2.1 (Figure 12) were well prepared and clean. Bleeding was controlled with dressing impregnated with epinephrine, and the retrograde cavities of both teeth were filled with MTA (ProRoot®, Dentsply, York, USA) (Figure 13). Once the filler material had set, the endoscope was used to confirm correct completion of the retrograde filling of 1.1 (Figure 14) and 2.1 (Figure 15). Regeneration of the bone defect was secured with beta-tricalcium phosphate particles (KeraOs-Keramat®, Santiago de Compostela, Spain) (Figure 16), and suturing was carried out with a non-reabsorbable 4/0 multifilament suture (Tevdek®, Teleflex®, Wayne, USA) (Figure 17).

**Figure 18:**
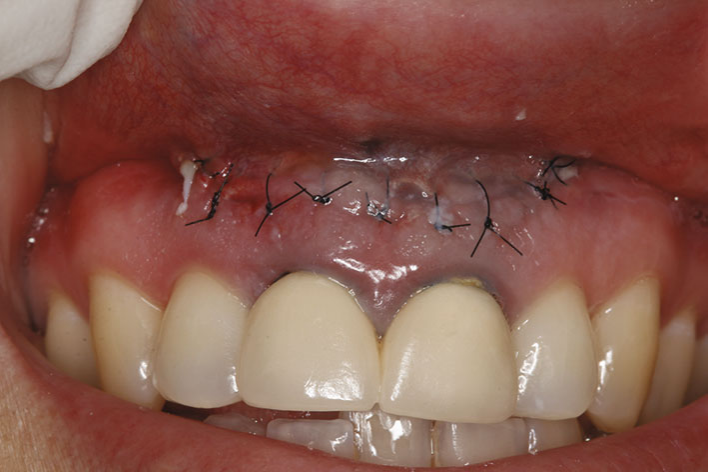
View after 7 days, before suture removal.

Amoxicillin 500 mg (Laboratorios Normon, Paterna, Spain) every 8 hours during 6 days was prescribed, along with ibuprofen 600 mg (Laboratorios Normon, Paterna, Spain) every 8 hours during three days and 0.12% chlorhexidine rinses (Perio Aid, Dentaid, Barcelona, Spain) every 8 hours during 7 days. The sutures were removed after one week (Figure 18). The histopathological study showed the presence of generally dense fibrocellular connective tissue fragments with chronic inflammatory foci.

**Figure 19:**
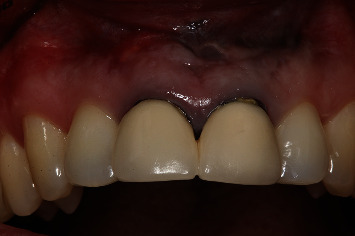
Clinical view one month after surgery: no suture dehiscence was noted.

**Figure 20:**
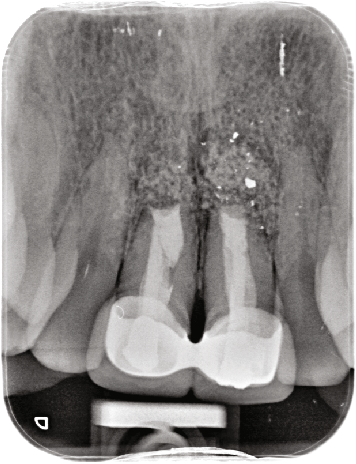
Periapical radiograph one month after surgery: correct retrograde cavity preparation is observed.

**Figure 21:**
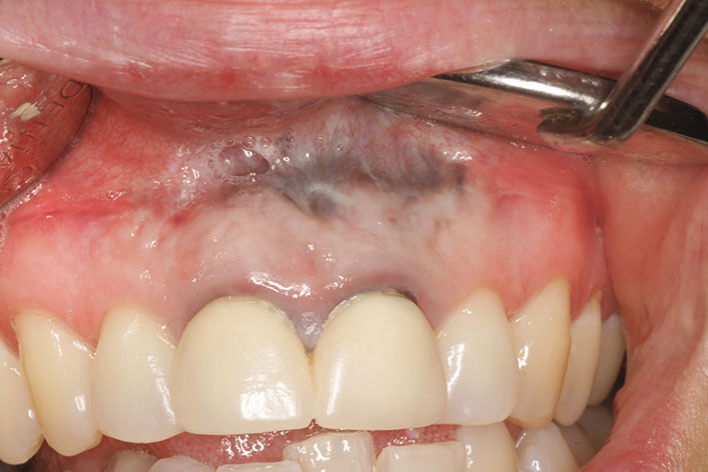
Clinical view 6 months after surgery, showing improvement of the soft tissues.

**Figure 22:**
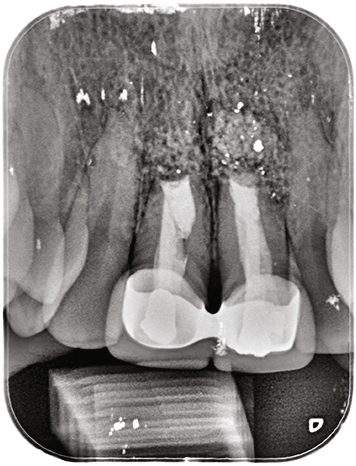
Periapical radiograph 6 months after surgery: correct progression of bone healing of the defect is observed.

**Figure 23:**
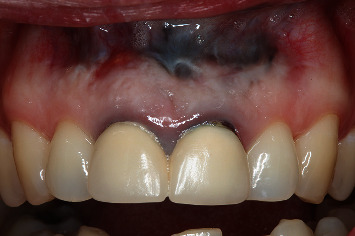
Clinical view one year after surgery: note the correct soft tissue healing.

**Figure 24:**
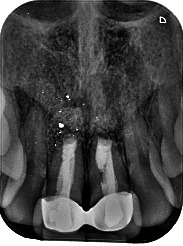
Periapical radiograph one year after surgery, showing complete bone regeneration of 1.1 and 2.1.

**Figure 25:**
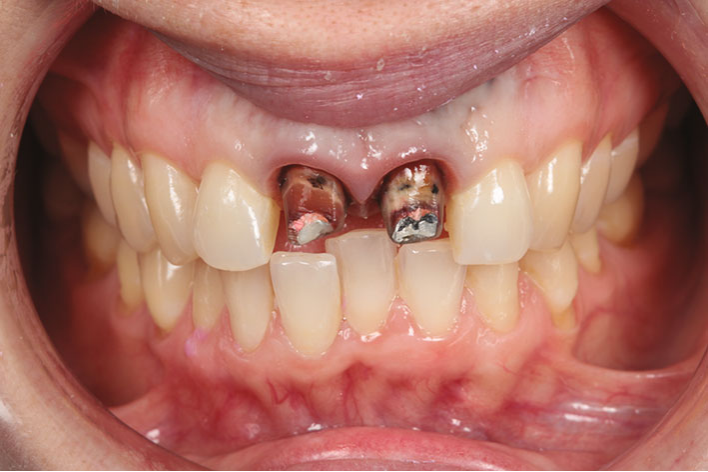
Frontal view of the stump of 1.1 and 2.1 based on the BOPT approach.

**Figure 26:**
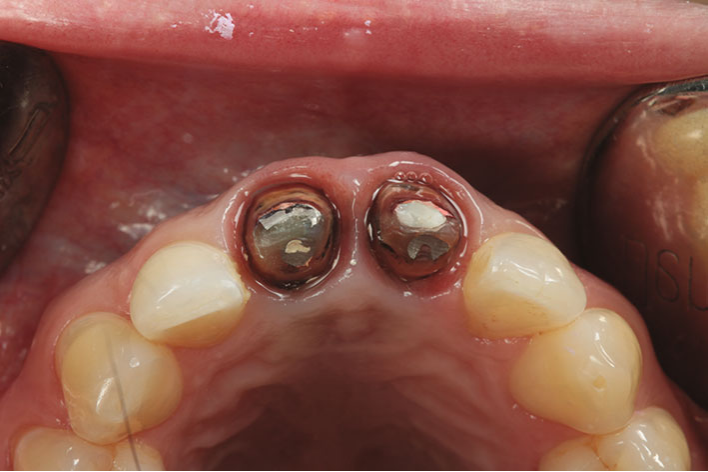
Occlusal view of the stump of 1.1 and 2.1 based on the BOPT approach.

**Figure 27:**
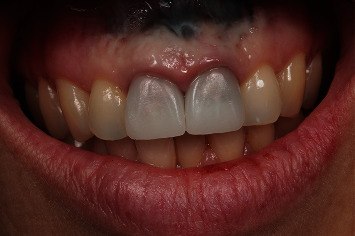
Fitting of the provisional crowns during four weeks to stabilize the surrounding gingival tissues.

**Figure 28:**
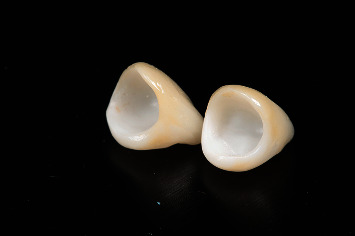
The definitive crowns made of monolithic zirconia with stratified feldspathic porcelain on the buccal aspect.

**Figure 29:**
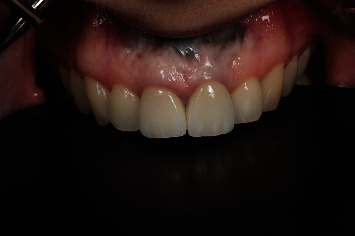
Clinical view after cementing the definitive crowns.

**Figure 30:**
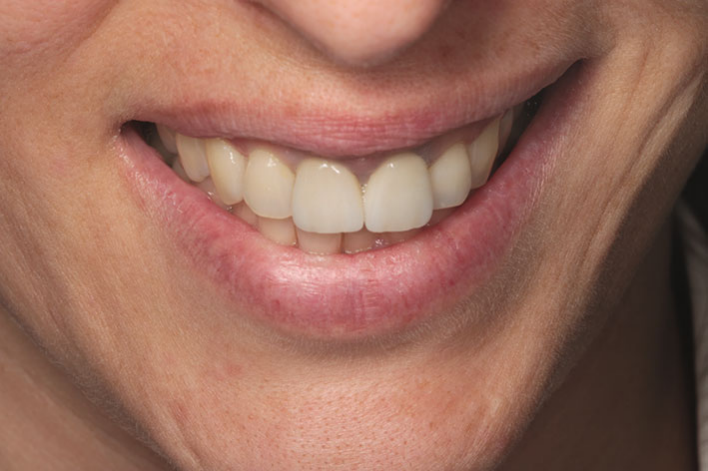
Extraoral view of smile after cementing the definitive crowns.

Clinical evaluation one month after surgery showed no suture dehiscences (Figure 19), and the periapical radiographic study confirmed correct retrograde filling (Figure 20). After 6 months, clinical improvement of the soft tissues was evidenced (Figure 21), with adequate progression of bone healing of the defect on the periapical radiographs (Figure 22). After one year, correct soft tissue healing was confirmed (Figure 23), with complete bone regeneration in the radiographic study and no radiolucencies (Figure 24). The patient was informed of the advisability of replacing the crowns of 1.1 and 2.1 because of marginal misadjustment and the associated negative aesthetic impact. Three years later, the patient accepted 5the proposed treatment. The old crowns were replaced with new ones, and the biologically oriented preparation technique (BOPT) was used, eliminating the finishing line in both the frontal view (Figure 25) and occlusal view (Figure 26). Provisional crowns were placed during four weeks to stabilize the gingival tissue surrounding the teeth (Figure 27). The definitive crowns were made of monolithic zirconia with stratified feldspathic porcelain on the buccal aspect (Figure 28), cemented with resin (RelyX Unicem, 3M®, Saint Paul, USA). Good periodontal health (Figure 29) and anterior aesthetic outcomes were achieved (Figure 30).

## 3. Discussion

In the present clinical case, periapical surgery was performed on two central upper incisors with persistent chronic periapical periodontitis that had already been subjected to periapical surgery 25 years ago, though without using the current magnification techniques. Over the years, conventional periapical surgery has evolved towards apical microsurgery, based on magnification and illumination of the surgical field with the endoscope and microscope [9–13]. The endoscope offers the surgeon excellent visibility [11], with an easier learning curve than the microscope, and both instruments afford a similar success rate [12]. The microscopic field is fixed, however, and the objective cannot be adjusted from different angles. Furthermore, in most cases, a mirror is needed for indirect visualization. In contrast, the endoscope offers easy adjustment, allowing visualization from different angles and providing direct images [13]. Endoscopy is therefore a versatile, rapid, and convenient magnification tool [12].

In addition to the introduction of ultrasound tips for retrograde cavity preparation [14], many materials have been used to secure correct cavity sealing, such as silver amalgam, zinc oxide-eugenol cements (IRM, Super-EBA), MTA, polycarboxylate cement (Durelon), or composites with dental adhesives (RetroPlast), among others.

At present, MTA is the most widely used material and with the strongest supporting evidence in the literature. A number of randomized clinical trials have compared MTA with other retrograde filler materials. Three such studies involving very low risk of bias [15–17] have reported success rates of 85-94.3% with the use of MTA. A meta-analysis published by Von Arx et al. [18] and a systematic review by Serrano-Giménez et al. [19] have defined MTA as the material of choice for retrograde cavity sealing in periapical surgery.

With regard to prosthetic rehabilitation, we chose the BOPT approach, involving elimination of the preexisting finishing line in the tooth stump, and rotary curettage of the gingival sulcus was performed using a diamond drill [20]—since a number of studies have evidenced improved periodontal tissue conditions of the teeth treated with this technique [21, 22].

## 4. Conclusions

In the reported patient with failed periapical surgical treatment of persistent chronic periapical periodontitis using the classical technique, the described periapical microsurgery approach with magnification and illumination of the surgical field was found to be effective, avoiding the need to extract the two central upper incisors.
